# Management of Stiffness following Total Knee Arthroplasty: International Survey on Surgeon Preferences

**DOI:** 10.1051/sicotj/2021008

**Published:** 2021-04-30

**Authors:** Karadi Hari Sunil Kumar, Georgios Mamarelis, Matthew Pettit, Vikas Khanduja

**Affiliations:** 1 Specialty Registrar in Trauma & Orthopaedics, Addenbrooke’s Hospital Hills Road Cambridge CB2 0QQ UK; 2 Specialty Registrar in Trauma & Orthopaedics, Royal London Hospital Whitechapel, London E1 1BB UK; 3 Medical Student, University of Cambridge, CB2 8PQ UK; 4 Consultant Orthopaedic Surgeon, Addenbrooke’s Cambridge University Hospital Hills Road Cambridge CB2 0QQ UK

**Keywords:** Total knee arthroplasty, Postoperative stiffness, Manipulation under anesthesia, Management, Outcomes

## Abstract

*Introduction*: Stiffness following total knee arthroplasty (TKA) is a challenging complication and can result in a poor functional outcome. There is considerable debate concerning the definition, work-up, and optimal management of this complication. The aim of this study was to record the definition of stiffness, management practices, and expectations of outcome among surgeons from an international community using a peer-reviewed questionnaire. *Methods*: A 23-item peer-reviewed online questionnaire was sent to all members of SICOT to gauge and record the management practices and expectations of outcome in the management of patients with stiffness following TKA. *Results*: A total of 315 surgeons completed this peer-reviewed questionnaire. Manipulation under anaesthesia (MUA) was the preferred treatment option for stiffness post-TKA, with a majority of the surgeons opting to carry out this procedure between 6 and 12 weeks following the index TKA. Physiotherapy and a continuous passive motion device were also used by the majority of surgeons following MUA, as additional treatment measures. *Discussion*: MUA is perceived to be a safe and effective primary treatment option for stiffness following TKA. It is best performed between weeks 6 and 12 with expected gains in range of motion from 10 to 20 degrees in 75% of patients.

## Introduction

Total knee arthroplasty (TKA) is being performed increasingly for treating end-stage arthritis of the knee [1]. The lifetime risk of undergoing a primary TKA for a population older than 25 years is 7% for males and 9.5% for females [1]. The goal of performing a TKA is to achieve a painless and stable knee joint with a functional range of motion (ROM) [2]. Patient satisfaction following TKA is influenced by a multitude of factors of which post-operative improvement in range of movement and meeting pre-operative patient expectation are key to achieving successful outcomes [3]. The 10-year implant survival rate is reported to be 96.1%, and the 20-year implant survival rate is 89.7% [4]. However, about 15–20% of the patients remain dissatisfied following the procedure and one of the reasons for this dissatisfaction is poor post-operative ROM [4]. A ROM of 10°–95° is required for walking and climbing stairs. Some activities of daily living require the knee joint to flex up to 115°, for example tying shoelaces while sitting [5], and some activities like sitting cross-legged and kneeling while praying require flexion beyond.

Post-operative stiffness is defined as an inadequate ROM causing functional limitations in activities of daily living and occurs in approximately 5–6% of TKAs. And some studies report an incidence of up to 20% [6–9]. However, caution is needed when interpreting these publications as ROM based definitions of stiffness used in the literature are inconsistent [6, 10, 11].

Stiffness following TKA can occur as a result of several conditions (Table 1) with different treatment options (Table 1). Arthrofibrosis is the primary cause of stiffness [2] and manipulation under anaesthesia (MUA) has been described as the first line of intervention from as early as 2 weeks to 4 months postoperatively [9]. However, there is no consensus for the optimal time to perform an MUA, whether MUA should actually be the first line of intervention and debate continues on post-MUA management of this difficult cohort of patients. Our group is keen on understanding these differences and has published on the subject previously [2, 12].

**Table 1:** Causes of stiffness following TKA and treatment options.

Causes of stiffness post-TKA	Treatment options available
Arthrofibrosis	Physiotherapy
Infection	Continuous passive motion (CPM) device
Implant malposition	Manipulation under anaesthesia
Dislocation of TKA	Arthroscopic arthrolysis
Fracture of polyethylene	Open arthrolysis
Peri-prosthetic fracture	Revision TKA

Currently, there is no clear definition of stiffness following TKA and a lack of a clear management algorithm, especially with the timing of MUA for stiffness. It is vital to understand the current management practices and expectations of outcomes concerning the management of stiffness following TKA across the international community. This would help surgeons have a better understanding of the management of this important problem.

The aim of the study was therefore to (1) define stiffness following TKA and work-up for this problem, (2) analyse the timing of MUA for stiffness following TKA, (3) identify the different modalities and duration of rehabilitation used following MUA, (4) assess the improvement in ROM after MUA and recurrence of stiffness, (5) ascertain complications of MUA experienced by surgeons, and (6) different patient-reported outcome measures (PROMs) used and patient satisfaction.

## Methods

A pilot survey was prepared following an in-house discussion of the research team addressing the study aims. This contained sections devoted to the surgeon’s current practice, including the diagnosis and investigation of stiffness, management options, rehabilitation protocol, and follow-up schedule. The questions were of a multiple-choice format, and the participants were encouraged to provide comments where necessary. The pilot survey was sent to an independent group of 20 senior knee arthroplasty surgeons in the UK, USA, Canada, and Australia. This process filtered ambiguities and the surgeons commented on the transparency and comprehensiveness of the questions, providing suggestions to improve the quality of the survey. The questionnaire was modified following the pilot survey and a final 23-item questionnaire-based survey (Supplementary Table 1s) was constructed and delivered through an online medium using Survey Monkey (*Survey Monkey®, San Mateo, California Office, One Curiosity Way, San Mateo, CA 94403*).

We chose SICOT as a target organization to undertake the survey because of its international profile with global representation. SICOT is an International Orthopaedic Organisation, governed by Belgian Law, with surgeon members from 110 member countries [13]. The online survey was delivered via e-mail to all SICOT members, which at the time of the survey were approximately 2500. A reminder email was sent to all SICOT members at 8 and 16 weeks following the initial email to improve the response rate. All data were stored anonymously on the survey monkey website with the facility to export the information to perform descriptive statistical analysis. This study received approval from the Institution’s Audit Department.

## Results

We received a response from 315 members of the organization after two reminders and that reflects the number of surgeons potentially interested in knee arthroplasty in the membership.

### Geographical location and experience of those completing the survey

**Figure 1:**
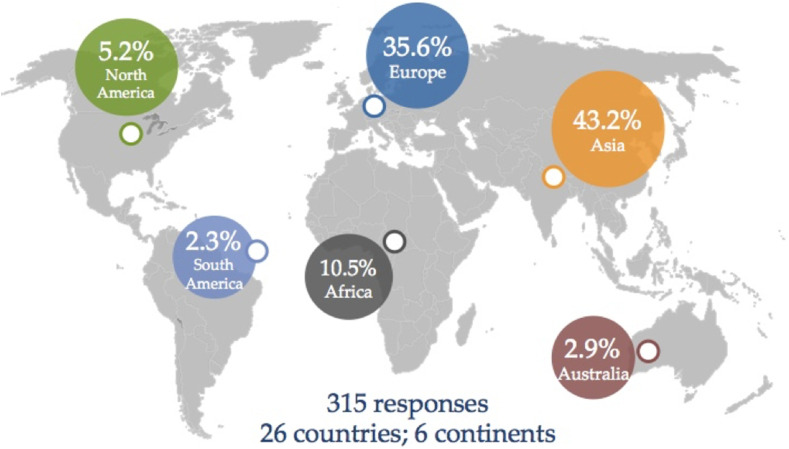
Survey participants by continent.

**Figure 2:**
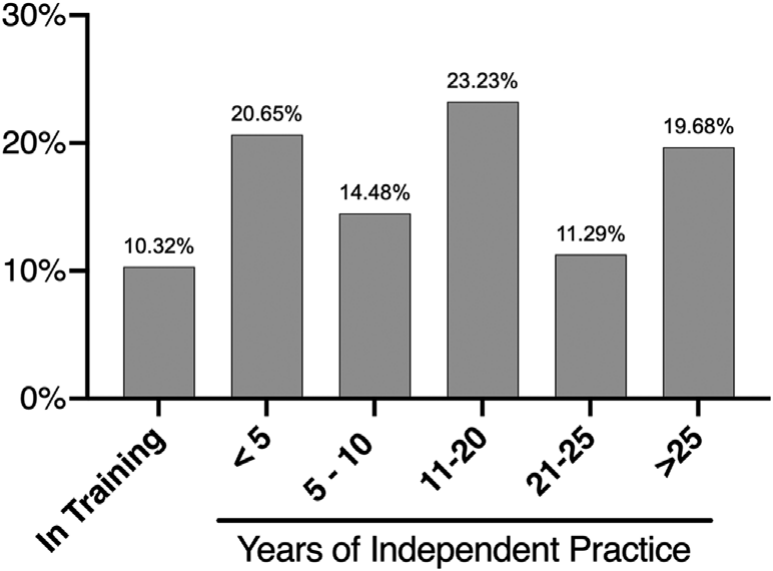
Results of the survey question: How many years have you been in independent practice?

**Figure 3:**
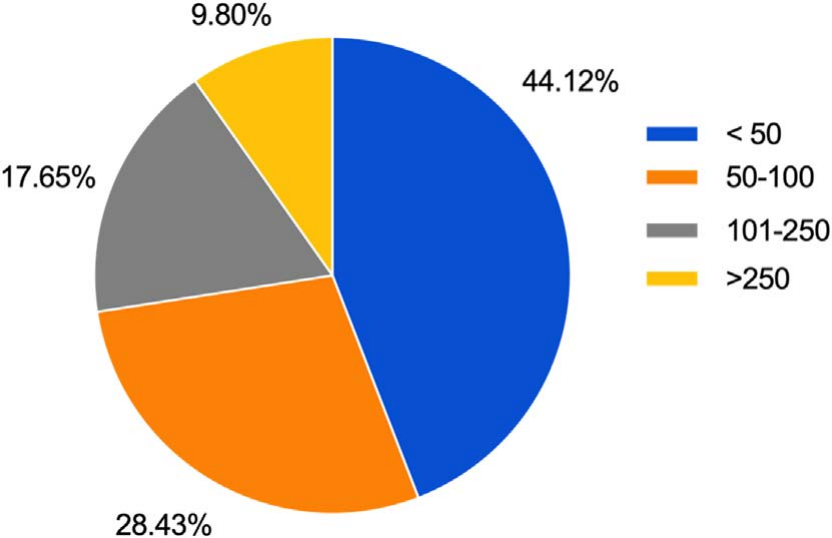
Results of the survey question: How many Total Knee Arthroplasties do you perform annually?

The responses received were from 26 countries across 6 continents. The majority of the responses were from Asia (43.2%) and Europe (35.6%). In addition, we received responses from Africa, North America, Australia, and South America (Figure 1). Forty percent of the responding surgeons were working in a teaching hospital. There was a wide range of experience in the respondents ranging from orthopaedic trainees to those with more than 25 years of independent practice (Figure 2). More than 55% of the participants performed 50 or more TKAs each year (Figure 3).

### Definition of TKA stiffness and work-up

**Figure 4:**
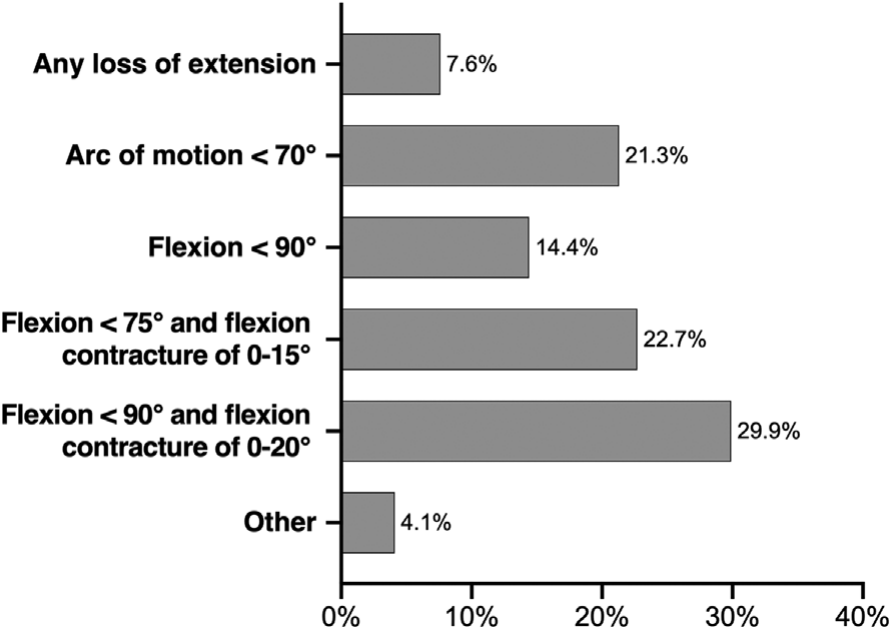
Results of the survey question: How do you define stiffness following Total Knee Arthroplasty?

Considerable variability was found among the participating surgeons in how they defined stiffness following TKA (Figure 4). There was no regional variability in the definition of stiffness. The majority of the participants (66.7%) perceived that less than 3% of patients developed a stiff knee post-operatively, while 19.5% felt that 3–5% developed stiffness following TKA. A majority (76.3%) also performed blood tests, including C-reactive protein (CRP) and Erythrocyte Sedimentation Rate (ESR), as a part of the investigations for stiffness etiology. In addition, 89.9% of the participants performed plain radiographs, 44.4% performed a Computed Tomography (CT) scan, and 14.9% performed Single Photon Emission Computed Tomography (SPECT) of the knee joint to rule out implant malposition and infection respectively.

### Timing of MUA

**Figure 5:**
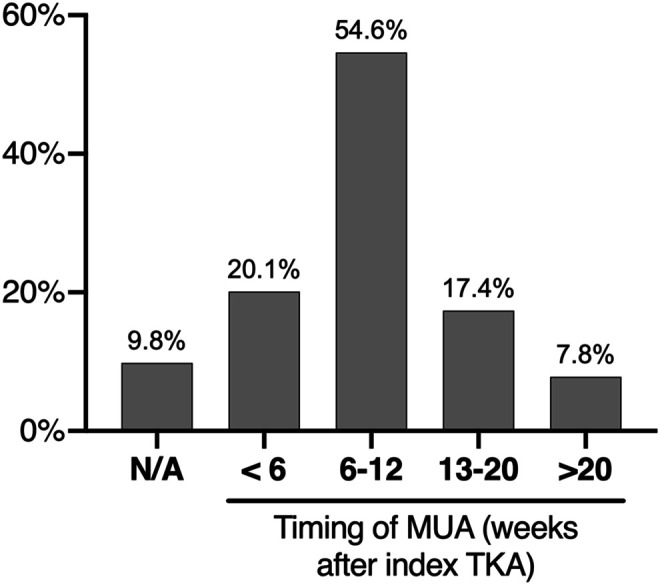
Results of the survey question: How long after the index TKA would you perform MUA? N/A indicates surgeons who would choose not to perform MUA for stiffness following TKA.

The majority of participating surgeons (90.2%) performed MUA for arthrofibrosis. With regards to the timing of MUA 54.6% considered that MUA for stiffness should be performed between 6 and 12 weeks following the index TKA, whereas 20.1% would perform MUA within the first 6 weeks, 17.4% between 12 and 20 weeks, and 7.8% would consider MUA after more than 20 weeks (Figure 5). A limited number (9.8%) would not perform MUA for stiffness following TKA.

### Rehabilitation following MUA

The majority (95.5%) of the participating surgeons offered physiotherapy to their patients following MUA; 60.7% of the surgeons offered physiotherapy routinely for 6 weeks and 28.2% for up to 3 months. Additionally, 71.7% of the participants used a Continuous Passive Motion (CPM) device, with a varied duration of use. CPM was used by 38.8% for inpatients until the achievement of the target ROM, 16.9% of the patients were discharged home with CPM if necessary, to achieve target ROM and 15.8% used CPM only for inpatients irrespective of achievement of ROM.

### Improvement in the ROM, recurrence rates, and treatment options

The participating surgeons who performed MUA for stiffness following TKA expected improvement in ROM, almost 75% of participants expected an improvement of a minimum of 10°–20°. A further sub-group analysis revealed that 31% of participants expected between 10° and 20° gain in the ROM, compared to 41% expecting 20°–30°, 14.8% expecting 30°–40° and 9.5% expecting >40° improvement in ROM following MUA.

The perceived recurrence rate of stiffness following MUA varied among participating surgeons: 32% estimated that the recurrence rate was less than 1%, 26% estimated that it was 1–5%, 18.6% estimated that it was 5–10%, and 23.3% estimated recurrence in more than 10% of cases.

In patients with recurrence of stiffness following MUA, nearly half (46.4%) of the participating surgeons elected not to perform a second MUA. Only 24.4% occasionally performed a second MUA and 17.7% would only perform a second MUA at the patient’s request. Thirty seven percent of the participants elected to combine MUA with arthroscopic arthrolysis, while 21.3% of participants would proceed to open arthrolysis and 12.9% would consider revision surgery if necessary. Additionally, 12.6% of the participants considered physiotherapy alone as appropriate management in case of a recurrence of stiffness.

### Complications following MUA

Manipulation under anaesthesia for stiffness following TKA was considered a safe procedure by the majority of participating surgeons, 71.9% estimated that the rate of complications including fracture, patella tendon rupture, and algodystrophy was less than 1%. The remainder of the participants estimated that their complication rates were 1–3% (19.4%) or more than 3% (8.7%).

### PROMs and patient satisfaction

A variety of scoring systems were used to assess TKA outcomes among the participating surgeons. The most popular scoring system was the Knee Society System (61.9%), followed by the Oxford Knee Score (28.6%). The majority of participants (60%) estimated that their patients are either satisfied or very satisfied with the outcome of MUA for stiffness following TKA, 32.5% estimated their patients to be neutral with regard to MUA outcome and 7.5% estimated that the patients would be dissatisfied with the outcome.

## Discussion

This is the first international survey that investigates the practices of orthopaedic surgeons concerning the diagnosis, investigation, and management of stiffness following TKA. We received responses from 315 orthopaedic surgeons, with varying experience, ranging from trainees to those with over 25 years of independent orthopaedic practice. The results revealed a wide variation in practice. Although 44.1% of the survey respondents performed less than 50 TKAs per year, the majority (55.9%) performed more than 50 TKAs per year including 9.8% who performed more than 250 TKAs per year (Figure 3). The participation of surgeons with a keen interest in TKA helped to improve the validity of these results.

We accept some limitations of our study. Firstly, electronic surveys are prone to a low response rate [14]. We chose this medium as this provided worldwide access and also afforded a quick response with a follow-up email reminder to complete the same. Our response rate was low with just over 12.6% of the total SICOT membership responding, although this still afforded sufficient numbers for data analysis and reflects the number of surgeons interested in knee arthroplasty in a generalist orthopaedic society. Secondly, the majority of the participants were from Asia and Europe (Figure 1), this may indirectly reflect the represent SICOT membership from these regions. Increased participation elsewhere globally may have revealed regional variations in practice if any. Thirdly, even though the questionnaire was comprehensive with 23-items, we were not able to capture all possible data due to the inherent limitation of survey-based research.

In this survey there was a wide variation in how participants defined stiffness following TKA, reflecting the breadth of definitions in the current literature [10]. In the future, it is recommended that the international consensus on the definition of stiffness achieved by The Knee Joint Fibrosis Working Group should be adopted which states a requirement for “a restricted ROM in flexion or extension, or both flexion and extension” in order to diagnose post-operative arthrofibrosis [11]. Additionally, the group has precisely graded the severity of arthrofibrosis into mild, moderate, and severe depending on either the restriction of extension (5°–10°, 11°–20°, > 20°) or the flexion range (90°–100°, 70°–89°, < 70°) [11]. The proportion of patients estimated to undergo MUA for stiffness following TKA ranges from 2.2% to 6% [15]. Our survey reinforced this with 86.3% of participating surgeons estimating that less than 5% of patients developed stiffness following TKA.

The optimal timing of MUA after TKA is controversial [19]. Some studies suggest that MUA within 12 weeks from TKA achieves better ROM outcomes compared with MUA performed after longer intervals [15, 16] one such study suggests that the therapeutic benefit is lost 26 weeks after TKA [16]. Other studies have established no difference in outcome following MUA performed before or after 12 weeks [17]. In our survey, 54.5% of the participating surgeons advised MUA within 6–12 weeks of TKA and 20.1% advised that MUA should be performed within 6 weeks of TKA, indicating that current surgical practice is to prefer MUA at generally shorter intervals (<12 weeks) from the index TKA.

Yercan et al. proposed that thorough investigations be performed prior to MUA to rule out infection, algodystrophy, or surgical error which are contraindicated for MUA [6]. Additionally, the Knee Joint Fibrosis Working Group suggests that a clinical diagnosis of joint fibrosis may be made after excluding other causes of stiffness, using investigations including plain radiographs, CT scans, serology, and aspiration [15]. Our survey demonstrated a similar sentiment, the most common investigations performed were blood tests, plain radiographs, and CT scans, which were preferred by 76.3%, 89.9%, and 44.6% of the participating surgeons respectively.

Issa et al. reported that the mean gain in flexion following MUA in their study was 33°, ranging from 5° to 65°, with a gain of 36.5° for early MUA compared to 17° for late MUA [16]. Our survey showed 41% of participants expected between 10° and 20° gain in ROM, with the remaining participants expecting greater and lesser gains in roughly equal proportions. There is no consensus on the usefulness of repeated MUA [18].

Additional use of CPM in combination with physiotherapy has been shown to be beneficial during the early rehabilitation phase following a primary TKA [18, 19]. Although a study from Boese et al. reported no clinically significant difference in ROM with the postoperative use of CPM [20]. A recent national survey from the UK reported that 68% of surgeons would routinely use CPM post manipulation [21]. A total of 72% of our study participants used CPM following MUA but for a varying duration similar to the UK practice, denoting that the CPM usage was similar across the globe. Post-operative physiotherapy following TKA is a key factor for maintaining ROM and therefore satisfactory outcomes [5, 6, 19]. Our survey reinforced this, as 89% of participants using physiotherapy for at least 6 weeks following MUA, and 27% using physiotherapy for 3 months.

There are four typical management options for stiffness following TKA. After MUA these are arthroscopic arthrolysis, open arthroscopic arthrolysis, and revision TKA [8, 10, 11]. Our survey showed that in recurrent stiffness following MUA, 36.8% of participants would perform MUA with arthroscopic arthrolysis and 21.3% would proceed directly to open arthrolysis. The Knee Joint Fibrosis Working Group has also suggested escalation along with these four treatment options [15].

Even though MUA is a non-invasive procedure, it is crucial to ensure that the benefits outweigh the risks and possible complications. MUA is perceived to be a safe procedure with 71.8% of participating surgeons estimating a complication rate of less than 1% following MUA. The complications for each of the procedure is listed in Table 2.

**Table 2:** Surgical options for stiffness and their possible complications.

Procedure	Complications
MUA	Bleeding
	Wound dehiscence
	Peri-prosthetic fracture of femur or tibia
	Dislocation of the TKR
	Fracture of the tibial post of polyethylene
	Rupture of patellar tendon
Arthroscopic arthrolysis	Infection
	Damage to bearing surface of TKR
	Damage to polyethylene insert
	Bleeding
Open arthrolysis	Infection
	Damage to bearing surface of TKR
	Damage to polyethylene insert
	Wound complication
Revision TKR	Infection
	Wound complication
	Recurrence of stiffness

### Strengths of the study

This is the first study to assess the management preferences of surgeons across the globe towards stiffness following TKA. The participating surgeons had different levels of experience both in terms of years in independent practice and the number of TKAs performed annually, which can be generalized to the whole orthopaedic community. In addition, the final questionnaire was devised following a preliminary validity assessment of the pilot survey by 20 senior knee arthroplasty surgeons from the International Community.

**Figure 6:**
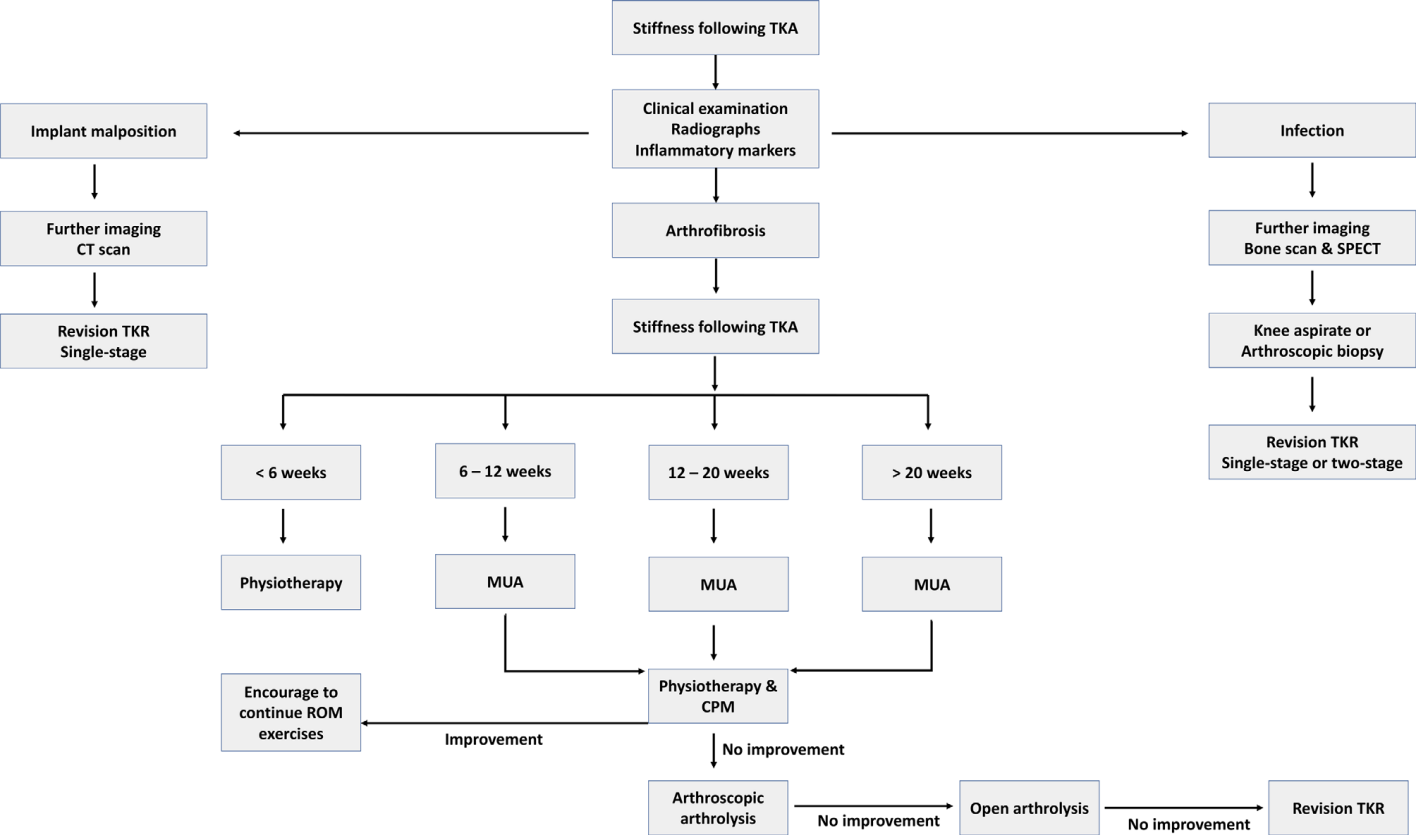
Algorithm for the treatment of stiffness post total knee arthroplasty.

A timely accurate diagnosis of stiffness following total knee arthroplasty is essential to appropriately manage the condition and ensure good patient satisfaction. A simplified treatment algorithm is shown in Figure 6, which applies to the majority of the cases presenting with stiffness following TKA.

## Conclusions

Stiffness is a well-recognized complication following TKA, which can result in a poor functional outcome. A thorough evaluation of patients with a stiff knee following TKA is essential to correctly diagnose arthrofibrosis as the cause of stiffness. MUA is widely recognized as the optimal first-line treatment to improve ROM in postoperative arthrofibrosis. Improvement in ROM of 10° to 20° can be expected in 75% of patients. Post-operative physiotherapy for 6–12 weeks is believed to be useful to maintain the gain in ROM. In cases where there is a recurrence of stiffness following MUA, our survey suggests a second MUA with or without arthrolysis should be considered. MUA is perceived as a safe procedure with a low complication rate, where a good functional outcome can be expected.

## Supplementary Material

The supplementary material of this article is available at https://www.sicot-j.org/10.1051/sicotj/2021008. ***Table 1s***. International Survey on Surgeon Preferences in the Management of Stiffness following Total Knee Arthroplasty.

## Conflict of Interest

All authors declare that they have no conflict of interest for the submitted work and have received no funding for this work.

KHSK – Chair of SICOT Young Surgeons Committee.

VK – Educational consultant Smith & Nephew & Arthrex, Chair SICOT Education Academy, President Elect British Hip Society, Past Chair – NAHR UK.
