# Supplementary material for: Management of Stiffness following Total Knee Arthroplasty: International Survey on Surgeon Preferences
Source: SICOT J. 2021 Apr 30;7:30. doi: 10.1051/sicotj/2021008 (PMC8086424; doi:10.1051/sicotj/2021008)
Supplement: Supplementary file 1 — Table 1s. International Survey on Surgeon Preferences in the Management of Stiffness following Total Knee Arthroplasty. [file sicotj-7-30-s1.pdf]

Supplementary Table 1

International Survey on Surgeon Preferences in the Management of Stiffness following Total Knee Arthroplasty.

1. **How many years have you been in independent practice?**
   - Currently in training
   - Less than 5 years
   - 5 to 10 years
   - 10 to 20 years
   - 20 to 25 years
   - More than 25 years
2. **How many Total Knee Arthroplasties do you perform annually?**
   - Less than 50 per year
   - 50 to 100 per year
   - 101 to 250 per year
   - >250 per year
3. **Where is your current practice located?**
   - North America
   - South America
   - Europe
   - Asia
   - Australia
   - Africa
4. **In what type of hospital do you practice?**
   - Academic or Teaching (University affiliated)
   - District General Hospital
   - Private Practice
   - Private Practice and Academic or Teaching (University affiliated)
   - Private Practice and District General Hospital
   - Other (please specify)
5. **How do you define stiffness following Total Knee Arthroplasty?**
   - Any loss of extension
   - Arc of motion < 70 degrees
   - Flexion < 90 degrees
   - Flexion < 75 degrees and Flexion contracture of 0 – 15 degrees
   - Flexion < 90 degrees and Flexion contracture of 0 – 20 degrees
   - Other (please specify)
6. **What percentage of your Total Knee Arthroplasties get stiffness?**
   - Less than 1%
   - 1-3%
   - 3-5%
   - More than 5%
   - Don't know
7. **What investigation/s do you perform to assess the cause of stiffness?(choose all that apply)**
   - FBC, ESR and CRP to rule out infection
   - Plain radiograph of the knee to assess alignment and size of the implant
   - CT Scan of the knee to assess malrotation
   - SPECT to rule our loosening and infection
   - MRI Scan
   - Other (please specify)

Supplementary Table 1

**8. What is your order of preference in terms of the type of intervention for the management of stiffness (without any obvious surgically correctable cause i.e. large implant) at 6 weeks following Total Knee Arthroplasty (TKA)? (Rank them from 1 to 6, 1 being the first choice, 2 the second choice and so on)**<br>Physiotherapy<br>Manipulation under Anaesthesia<br>MUA + Arthroscopic Arthrolysis<br>Open Arthrolysis<br>Revision Total Knee Arthroplasty<br>No treatment
**9. At what time after the index TKA would you consider Manipulation Under Anaesthesia (MUA) for stiffness?**<br>6 weeks or less<br>More than 6 weeks up to 12 weeks<br>More than 12 weeks up to 20 weeks<br>More than 20 weeks
**10. Considering you perform MUA for stiffness after TKA, how late after the index operation would you perform MUA if needed?**<br>Up to 12 weeks<br>Up to 20 weeks<br>Up to 26 weeks (6 months)<br>Up to 52 weeks (12 months)<br>Anytime<br>No - I don't perform MUA<br>Other (please specify)
**11. How much average improvement in the Range of Movement (ROM) do you expect following a manipulation under anaesthesia?**<br>10 to 20 degrees<br>21 to 30 degrees<br>31 to 40 degrees<br>More than 40 degrees<br>Other (please specify)
**12. Do you use a Continuous Passive Motion (CPM) device after MUA for stiffness?**<br>Yes – CPM as inpatient until target ROM achieved<br>Yes – Patients go home with CPM until target ROM achieved<br>Yes – CPM only as inpatient irrespective of ROM achieved<br>No - I don't use CPM
**13. Do you routinely offer Physiotherapy after MUA?**<br>Yes - for 6 weeks following MUA<br>Yes - for 3 months following MUA<br>Yes - for 6 months following MUA<br>No - I do not offer Physiotherapy following MUA
**14. Do your patients receive a femoral nerve catheter (to provide continuous infusion of local anaesthesia) for pain relief following a MUA? If yes, for how long?**<br>Yes – for less than 1 day<br>Yes – for 1 to 3 days<br>Yes – for 3 to 5 days<br>Yes – for more than 5 days<br>No – I don't use nerve catheter
**15. When you perform MUA for a stiff TKA, do you inject the joint with any of the following?**<br>Local Anaesthetic<br>Steroid<br>Anti-inflammatory<br>Local anaesthetic + Steroid + Anti-inflammatory<br>No drug injected into the joint<br>Other (please specify)

Supplementary Table 1

16. What is the rate of recurrence of stiffness following a MUA in your practice?

<1%
1-5%
5-10%
Over 10%

17. Would you consider a second MUA if one of your patients had a recurrence of stiffness?

Yes – always
Yes – sometimes
Yes – rarely if patient requests
No - I do not perform a second MUA
No - I never perform MUA

18. If the MUA that you have performed for stiffness is not successful, what would you consider as your next step?

Another MUA
Physiotherapy only
MUA and Arthroscopic Arthrolysis
Open Arthrolysis
Revision Surgery
No treatment
Other treatment (please specify)

19. What is the rate of complications following a MUA for a stiff TKA in your practice (Fracture, CRPS, Patella tendon rupture etc)?

<1%
1-3%
3-5%
5-10%
>10%

20. What scoring system/s do you use to assess the outcome of any intervention for a stiff knee following TKA? (choose all that apply)

Knee Society Score
KOOS - Knee Injury and Osteoarthritis Outcome Score
Oxford Knee Score
EQ5D
VAS
SF36
WOMAC - Western Ontario and McMaster University Osteoarthritis Index
Other

21. How satisfied are the patients with the MUA for a stiff TKA in your practice?

Very Satisfied
Satisfied
Neutral
Dissatisfied
Very dissatisfied
Other (please specify)

22. How likely is it that you would recommend MUA for stiffness after TKA to a friend or colleague?

Not at all likely – 0; Extremely likely – 10

1	2	3	4	5	6	7	8	9	10

23. Kindly let us know of your comments or suggestions regarding this survey on MUA for stiffness following TKA.

Blank space for comments
